# Associations among physical literacy, physical activity, and well-being in South Korean university students: a structural equation modeling study

**DOI:** 10.3389/fspor.2026.1828300

**Published:** 2026-04-14

**Authors:** Seungwoo Choi, Ansu Lee

**Affiliations:** Department of Physical Education, Kyungpook National University, Daegu, Republic of Korea

**Keywords:** physical literacy, physical activity, well-being, university students, structural equation modeling

## Abstract

**Background:**

Physical literacy has been increasingly recognized as a foundational capability that supports lifelong engagement in physical activity and positive health outcomes. However, the structural relationships among PL, PA, and well-being remain insufficiently examined in university populations. This study investigated the direct and indirect associations among physical literacy, physical activity, and well-being in South Korean university students.

**Methods:**

A cross-sectional survey was conducted with 361 undergraduate students. Physical literacy was measured using the Perceived Physical Literacy Instrument, physical activity was assessed using the International Physical Activity Questionnaire–Short Form, and well-being was measured using the Mental Health Continuum–Short Form. Confirmatory factor analysis and structural equation modeling were performed to examine the hypothesized relationships. Indirect effects were tested using bootstrapping procedures.

**Results:**

Physical literacy showed a significant association with physical activity (*β* = 0.49, p < 0.001) and well-being (*β* = 0.62, *p* < 0.001). However, physical activity was not significantly associated with well-being (*β* = 0.03, p *=* 0.548), and the indirect pathway from physical literacy to well-being through physical activity was not significant (*β* = 0.02, *p* = 0.655).

**Conclusions:**

Physical literacy appears to function not only as a correlate of physical activity but also as a psychosocial resource associated with well-being among university students. Promoting physical literacy in higher education settings may support students' psychological health and active lifestyles.

## 1 Introduction

The undergraduate years represent a distinctive phase of the transition to adulthood and university life characterized by substantial physical, psychological, and social changes. During this period, students must adapt to increased academic demands, reorganize social relationships, and manage greater autonomy within a relatively unstructured environment. At the same time, the transition from secondary education to university is often accompanied by the removal of compulsory or highly structured physical activity (PA), placing greater responsibility on students' self-regulation, motivation, and perceived competence to maintain active lifestyles (1). Recent evidence indicates that PA levels often decline during the university years, while vulnerability to psychological distress and reduced subjective well-being (WB) increases (2, 3), highlighting university students as a population at elevated health risk.

Unlike young adults more broadly, university students constitute a distinct and policy-relevant population whose health behaviors are shaped by specific institutional and environmental contexts. Universities may provide an important institutional context for health promotion during the transition to adulthood (4). From both educational and health-promotion perspectives, identifying factors that support sustained PA participation and WB in this population is therefore of considerable importance.

Physical literacy (PL) has emerged as a comprehensive framework for understanding lifelong engagement in PA and its broader health implications (5). PL is commonly defined as the motivation, confidence, physical competence, and knowledge required to value and participate in PA throughout the life course (6). Conceptually, PL extends beyond behavior-focused models by emphasizing the internal capacities that enable individuals to initiate, adapt, and sustain active lifestyles across diverse contexts (7, 8). In this sense, PL has been discussed in relation to perspectives such as embodied cognition, lifespan development, and self-determination-related approaches to motivation and engagement (7–10). These attributes may be particularly salient in autonomy-driven environments such as universities, where external structures supporting PA are reduced and individual agency becomes central to health-related decision-making.

A substantial body of empirical research has demonstrated positive associations between PL and PA across different age groups, including adolescents and young adults (11–14). Individuals with higher levels of PL are more likely to possess the motivational orientation, perceived competence, and contextual understanding necessary to engage in regular PA, even in the absence of external requirements. Accordingly, PL has often been conceptualized as a proximal correlate of PA behavior and a potential foundation for sustained participation (7, 15). At the same time, extensive evidence has documented beneficial associations between PA and mental health outcomes, including emotional and social WB (3, 16).

However, emerging research indicates that the contribution of PL to mental health may not be fully explained by PA behavior alone. Increasing attention has been directed toward the possibility that PL is associated with WB through psychosocial pathways independent of current PA levels (17, 20). The affective and cognitive dimensions of PL, such as confidence, enjoyment, perceived meaning, and understanding of movement, conceptually overlap with psychological resources associated with WB, including autonomy, self-efficacy, and resilience (21–23). In this sense, PL may function not only as a behavioral precursor but also as a broader psychosocial resource that supports adaptive functioning and psychological flourishing.

Evidence supporting this perspective has accumulated among university student populations in East Asian and comparable sociocultural contexts. Studies have reported significant associations between PL and mental health-related outcomes, including subjective WB, resilience, and quality of life (17, 19, 24). Nevertheless, relatively few studies have examined PL, PA, and WB within a single structural model, and such evidence remains limited in Korean university populations.

Accordingly, the present study aimed to investigate the structural relationships among PL, PA, and WB in South Korean university students. Specifically, this study examined whether PL was associated with WB directly, indirectly through PA, or both, using a structural equation modeling (SEM) approach. By addressing these questions, this study seeks to contribute to the growing literature on PL and mental health and to provide evidence relevant to student health promotion in higher education settings.

## 2 Materials and methods

### 2.1 Research design and hypotheses

**Figure 1:**
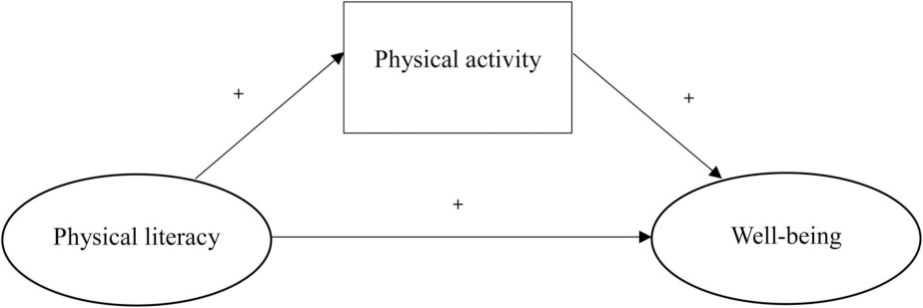
Hypothesized model of the relationships among physical literacy, physical activity, and well-being.

This study employed a cross-sectional, quantitative design using SEM to test the theoretically grounded relationships among PL, PA, and WB. Based on previous theoretical and empirical evidence, the proposed model assumes that PL influences WB both directly and indirectly through PA (Figure 1).

Accordingly, the following hypotheses were proposed:

H_1_: Physical literacy (PL) positively affects physical activity (PA) among Korean university students.
H_2_: Physical activity (PA) positively affects well-being (WB) among Korean university students.
H_3_: Physical literacy (PL) positively affects well-being (WB) among Korean university students.
H_4_: Physical literacy (PL) indirectly affects well-being (WB) through physical activity (PA) among Korean university students.

### 2.2 Procedure and participants

This study was reviewed and approved by the Institutional Review Board of Kyungpook National University (approval no. 2024-0210). All procedures adhered to the ethical principles of the Declaration of Helsinki, and data collection commenced only after ethical approval was obtained. The participants were undergraduate students enrolled at K University in Daegu, South Korea.

Data were collected using an online questionnaire in May 2024. Recruitment announcements were distributed through university bulletin boards and social media platforms. Before beginning the survey, participants were provided with an information sheet outlining the purpose of the study, the voluntary nature of participation, and the assurance of anonymity and confidentiality. Participants provided informed consent electronically before accessing the questionnaire. The survey took approximately 15–20 min to complete and included validated instruments for measuring PL, PA, and WB. Participants were informed that they could withdraw at any point without penalty, and no incentives were offered.

A total of 397 undergraduate students initially participated in the survey. Incomplete responses were defined as questionnaires with missing data on any of the main study variables (PL, PA, or WB). Inattentive responses were screened using predefined data-quality criteria, including uniform response patterns across Likert-type items, unusually short completion times, and repetitive response sequences across consecutive items. These criteria were applied to reduce the influence of careless or insufficient-effort responding. After excluding 36 incomplete or inattentive responses based on these criteria, the final analytic sample comprised 361 students (199 male, 55.1%; 162 female, 44.9%).

With respect to academic year, 169 participants (46.8%) were first-year students, 77 (21.3%) were second-year students, 68 (18.8%) were third-year students, and 47 (13.0%) were fourth-year students. Regarding academic major, 109 students (30.2%) were enrolled in humanities and social sciences, 147 (40.7%) in engineering, and 105 (29.1%) in natural sciences. Detailed demographic characteristics of the participants are presented in Table 1.

**Table 1:** General characteristics of the participants (*n* = 361).

Characteristic	Category	*n*	%
Gender	Male	199	55.1
	Female	162	44.9
Academic year	First year	169	46.8
	Second year	77	21.3
	Third year	68	18.8
	Fourth year	47	13.0
Academic major	Humanities and social sciences	109	30.2
	Engineering	147	40.7
	Natural sciences	105	29.1

### 2.3 Measures

#### 2.3.1 Measurement of physical literacy

PL was measured using the Perceived Physical Literacy Instrument (PPLI) developed by Sum et al. (25) and adapted into Korean by Choi and Lee (26). The PPLI comprises nine items across three subfactors: sense of self and self-confidence (3 items; e.g., “I am confident in my ability to participate in physical activities”), self-expression and communication with others (3 items; e.g., “I can communicate effectively with others through physical activities”), and knowledge and understanding (3 items; e.g., “I understand the benefits of regular exercise”). All items are rated on a 5-point Likert scale (1 = strongly disagree, 5 = strongly agree), with higher scores indicating greater PL.

In the present study, an overall PL score was calculated by averaging all nine items, consistent with prior applications of the PPLI. Subscale scores were not analyzed separately, as the primary focus of this study was on overall perceived PL as a holistic construct. All subfactors demonstrated acceptable internal consistency, with Cronbach's *α* values meeting the recommended threshold of 0.70.

#### 2.3.2 Measurement of physical activity

PA was assessed using the Korean version of the International Physical Activity Questionnaire-Short Form (IPAQ-SF) and its Korean validate version (27, 28). The IPAQ-SF comprises seven items assessing vigorous-intensity PA (2 items), moderate-intensity PA (2 items), walking (2 items), and sitting time (1 item) during the previous seven days. Participants reported both the number of days and average duration of each activity type, and only activity bouts lasting at least 10 min were included in the analysis.

Following the official IPAQ scoring protocol, total PA volume was calculated as metabolic equivalent task minutes per week (MET-min/week) by summing MET-adjusted activity time across walking, moderate-, and vigorous-intensity activities (walking = 3.3 METs, moderate-intensity PA = 4.0 METs, vigorous-intensity PA = 8.0 METs). Because raw MET-min/week values were positively skewed, log-transformed values were used in the SEM analysis. Previous studies have reported acceptable reliability and validity for both the IPAQ-SF (Spearman's *ρ* ≈ 0.80) (27), and the Korean version (Spearman's *ρ* = 0.427–0.646; k = 0.365–0.620) (28).

#### 2.3.3 Measurement of well-being

WB was measured using the Mental Health Continuum-Short Form (MHC-SF) developed by Keyes (29) and validated in Korean by Lim et al. (30). The MHC-SF comprises 14 items across three domains: emotional WB (3 items; e.g., “During the past month, how often did you feel happy?”), social WB (5 items; e.g., “How often did you feel that you belonged to a community?”), and psychological WB (6 items; e.g., “How often did you feel that your life has a sense of direction or meaning?”). All items are rated on a 6-point Likert scale (0 = not at all, 5 = every day), with higher scores reflecting greater WB. In this study, an overall WB score was calculated by averaging all 14 items of the MHC-SF, and domain-specific scores were not analyzed separately. The MHC-SF demonstrated strong internal consistency, with all subdomains meeting the recommended reliability criterion (Cronbach's *α* ≥ 0.70), indicating satisfactory measurement relaiblity.

### 2.4 Data analysis

All statistical analyses were conducted using SPSS version 27.0 (IBM Corp., Armonk, NY, USA) and AMOS version 26.0 (IBM Corp). Descriptive statistics (mean, standard deviation, skewness, and kurtosis) were computed to examine the data distribution and variable characteristics. Normality was evaluated according to the criteria of absolute skewness ≤ 3 and kurtosis ≤ 7 (31, 32). Internal consistency reliability was assessed using Cronbach's *α*. Pearson's correlation analysis was conducted to examine the associations between the main study variables (PL, PA, and WB).

To evaluated the measurement structure, confirmatory factor analysis (CFA) using maximum likelihood estimation was conducted prior to structural model testing. PL and WB were specified as second-order latent constructs. For PL, three first-order latent indicators were modeled: sense of self- and self-confidence, self-expression and communication with others, and knowledge and understanding. For WB, three first-order latent indicators were modeled: emotional WB, social WB, and psychological WB. In contrast, PA was treated as an observed variable represented by log-transformed total MET-min/week derived from the IPAQ-SF.

Model fit was evaluated using *χ*²/df, RMR, GFI, AGFI, NFI, TLI, CFI, and RMSEA, with 90% confidence intervals. Convergent and discriminant validity were examined through average variance extracted (AVE) and composite reliability (CR). Subsequently, SEM was used to test the hypothesized direct and indirect associations among PL, PA, and WB. Indirect effects were examined using bias-corrected bootstrapping with 2,000 resamples, and statistical significance was evaluated based on both *p*-values and bias-corrected 95% confidence intervals (CIs).

## 3 Results

### 3.1 Descriptive statistics

Descriptive statistics for PL, PA, and WB are presented in Table 2. The PA variable represents log-transformed total PA volume, expressed as Log(MET-min/week), derived from the IPAQ-SF. Accordingly, the reported mean values should be interpreted as a transformed indicator of total PA volume rather than as raw activity minutes. Skewness and kurtosis values were within acceptable ranges for all variables (±3 for skewness and ±7 for kurtosis), indicating that the assumption of normality was satisfied (31, 32).

**Table 2:** Descriptive statistics and correlations (*n* = 361).

Variable	Mean	SD	Skewness	Kurtosis	1	2	3
1. Physical Literacy	3.66	0.59	−0.23	−0.08	−		
2. Physical Activity^a^	7.70	0.74	−0.22	0.28	0.409***	−	
3. Well-being	2.56	0.99	0.19	−0.40	0.504***	0.240***	−

a Data were log-transformed and are presented as ln(MET-min/week).

*** *p* < 0.001.

PL was positively correlated with PA (*r* = 0.41, *p* < 0.001) and WB (*r* = 0.50, *p* < 0.001), and PA was positively correlated with WB (*r* = 0.24, *p* < 0.001). All correlation coefficients were below 0.80, suggesting that multicollinearity was not a concern (31, 32).

### 3.2 CFA measurement model

A second-order CFA was conducted to validate the measurement structure of PL and WB prior to SEM analysis. In the measurement model, PL was specified as a second-order latent construct indicated by three first-order factors: sense of self and self-confidence, self-expression and communication with others, and knowledge and understanding. WB was also specified as a second-order latent construct indicated by emotional, social, and psychological WB. Pa was not included as a latent construct because it was treated as an observed variable using long-transformed total MET-min/week.

The PPLI demonstrated a clear three-factor structure comprising sense of self and self-confidence, self-expression and communication with others, and knowledge and understanding*.* All standardized loadings exceeded 0.50 (*p* < 0.001), supporting the adequacy of the factor structure and retention of all items in the final model. The model demonstrated good fit [*χ*²/df = 2.279, RMR = 0.024, GFI = 0.966, AGFI = 0.937, NFI = 0.949, TLI = 0.956, CFI = 0.971, RMSEA = 0.060, 90% CI (0.039, 0.081)]. Convergent validity was confirmed (AVE = 0.868, CR = 0.951), and the AVE for each factor exceeded the squared correlations among the subfactors, supporting discriminant validity.

The MHC-SF also supported a three-factor model encompassing emotional, social, and psychological WB*.* The initial model demonstrated marginal fit [*χ*²/df = 3.057, RMR = 0.078, GFI = 0.910, AGFI = 0.873, RMSEA = 0.076, 90% CI (0.064, 0.087)]. To improve model fit, a limited number of residual covariances were added between items within the same latent factor, guided by modification indices and theoretical considerations. These refinements were justified by the conceptual similarity and shared wording of the involved items, and no changes were made to the underlying factor structure or item loadings. Subsequently, the model demonstrated substantially improved fit [*χ*²/df = 2.197, RMR = 0.067, GFI = 0.943, AGFI = 0.914, NFI = 0.953, TLI = 0.965, CFI = 0.974, RMSEA = 0.058, 90% CI (0.045, 0.070)]. Convergent (AVE = 0.698, CR = 0.873) and discriminant validity were supported, confirming the internal consistency and distinctiveness of each subfactor.

### 3.3 SEM results for the structural model and hypotheses testing

After validating the measurement model, SEM was constructed to examine the hypothesized relationships between PL, PA, and WB. The initial model exhibited suboptimal fit [*χ*²/df = 3.699, RMSEA = 0.087, 90% CI (0.060, 0.115)], indicating that refinement was needed. Other indices (CFI = 0.964, TLI = 0.937, GFI = 0.965, AGFI = 0.918) were acceptable, suggesting that the structure had partial adequacy. A covariance between the residuals of emotional and social WB was specified to reflect their theoretically related yet distinct facets within the MHC-SF framework (29, 30), which has been observed empirically, including in Korean validation studies. This specification is consistent with the MHC-SF framework, in which emotional and social WB are theoretically distinguishable yet empirically correlated facets of positive mental health, and such correlations have also been observed in the Korean validation studies of MHC-SF (29). The model fit improved substantially following this adjustment [*χ*²/df = 2.904, RMR = 0.038, GFI = 0.975, AGFI = 0.936, NFI = 0.965, TLI = 0.956, CFI = 0.977, RMSEA = 0.073, 90% CI (0.056, 0.089)], confirming that the refined model provided a valid and parsimonious representation of the interrelationships among PL, PA, and WB.

**Figure 2:**
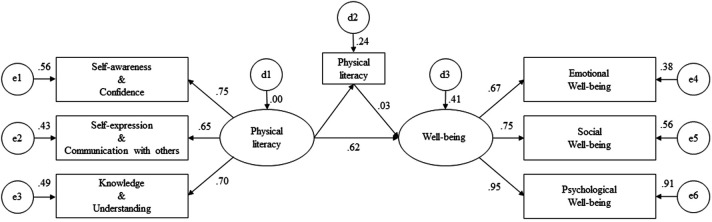
Final structural equation model illustrating standardized path coefficients among physical literacy, physical activity, and well-being.

The hypothesized relationships among PL, PA, and WB were examined using structural path analysis (Figure 2). PL showed a significant positive association with PA (*β* = 0.49, *p* < 0.001) and WB (*β* = 0.62, *p* < 0.001), indicating that students with higher PL tended to report greater PA and higher WB. In contrast, PA was not significantly associated with WB (*β* = 0.03, *p* = 0.548). The indirect pathway from PL to WB through PA was also not significant (*β* = 0.02, *p* = 0.655). This finding indicates that PA did not mediate the association between PL and WB. However, the total effect of PL on WB remained significant (*β* = 0.60, *p* < 0.001), suggesting that PL was associated with WB primarily through a direct rather than an indirect pathway. Table 3 presents the results of the estimated structural path analysis.

**Table 3:** Structural path analysis results.

Path		B	β	SE	CR	*p*-value
H_1_	PL → PA	0.84	0.49	0.11	7.75	<0.001
H_2_	PA → WB	0.03	0.03	0.05	0.60	0.548
H_3_	PL → WB	0.99	0.62	0.16	6.38	<0.001
H_4_	Indirect (PL → PA → WB)	0.03	0.02	0.06	-	0.655
Total Effect (PL → WB)		0.97	0.60	0.13	-	<0.001

B Unstandardized Estimate, β Standardized Estimate, SE Standard Error, CR Critical Ratio, PL Physical Literacy, PA Physical Activity, WB Well-Being.

Indirect effects were tested using a bias-corrected bootstrap procedure with 2,000 resamples, and statistical inference was based on both *p*-values and bias-corrected 95% confidence intervals.

## 4 Discussion

The present study examined the structural relationships among PL, PA, and WB in a sample of South Korean university students. The findings indicate that PL was positively associated with both PA and WB, whereas PA was not significantly associated with WB in the structural model. These results suggest that PL may be more closely associated with WB through direct psychosocial pathways than through PA participation alone. Previous studies have also examined whether PA mediates the relationship between PL and WB, with findings indicating that the mediating role of PA may vary depending on population characteristics and contextual conditions (33). In this regard, PL may be understood not only as a correlate of active behavior but also as a broader psychological resource associated with WB during emerging adulthood.

From a theoretical perspective, the findings support the view of PL as a multidimensional construct encompassing affective, cognitive, and behavioral attributes related to movement engagement (5, 7–9). PL has often been conceptualized as a foundation for sustained participation in PA because individuals with higher levels of PL tend to possess the motivation, confidence, and understanding necessary to engage in movement across diverse contexts (7, 9). Consistent with this perspective, the present results indicate that students with higher PL reported higher levels of PA, supporting previous studies that identify PL as an important correlate of PA participation among adolescents and young adults (11–14).

Importantly, PL showed a significant direct association with WB even after accounting for PA in the structural model. This finding suggests that the relationship between PL and WB may not be fully explained by behavioral engagement in PA alone. Rather than reflecting only readiness for activity, PL may capture psychological and experiential attributes that are closely related to WB, such as confidence, self-perception, autonomy, and meaningful engagement in movement contexts (17, 18, 24). In this sense, PL may be associated not only with PA participation but also with broader psychosocial resource relevant wo WB among university students (7, 18).

The positive association between PL and PA observed in this study is consistent with previous research demonstrating that individuals with higher PL are more likely to participate in regular PA (11, 12, 19). Recent evidence among university students also suggests that PL is positively associated with PA participation and adaptive psychological characteristics such as positive self-esteem (34). Although the present study focused on overall perceived PL rather than individual subdomains, PL-related attributes such as confidence in movement abilities, positive attitudes toward activity, and understanding of the value of exercise may help explain why students with higher PL tend to report greater participation in PA. These characteristics may be particularly important in university settings, where PA often depends less on formal requirements and more on students' self-directed choices and perceived competence.

In contrast, the non-significant association between PA and WB differs from a substantial body of literature reporting positive relationships between PA and mental health outcomes (3, 35). One possible explanation is that total activity volume alone may not fully capture the qualitative aspects of PA that are relevant to WB, such as enjoyment, autonomy, social interaction, and perceived competence. In university populations, the relationship between PA and psychological WB may therefore depend not only on the amount of activity performed but also on the context and quality of movement experiences, as well as psychosocial factors such as perceived social support, satisfaction of basic psychological needs, and physical self-efficacy (35, 36). Accordingly, the present findings may indicate that PA volume alone is insufficient to explain variation in WB in this population.

Another possible explanation relates to the broader development and contextual conditions experienced by university students. During emerging adulthood, students often face academic pressures, career uncertainty, and social transitions that may influence psychological WB independently of PA levels (2). Under such circumstances, PL-related psychological resources, such as confidence, perceived competence, and constructive engagement with movement, may show a closer association with WB than activity volume alone. Therefore, the absence of a significant PA–WB pathway in this study should not be interpreted as evidence against the potential mental health benefits of PA, but rather as suggesting that the relationship may be more conditional and context-dependent than a simple volume-based indicator can capture.

Taken together, the present findings suggest that PL may represent an important psychological and behavioral resource for university students (7, 18). Rather than viewing PL solely as a precursor to PA, it may be useful to consider PL as a broader capability that shapes how individuals perceive, value, and engage with movement experiences (7, 37). From this perspective, PL reflects an ongoing process of individual development through meaningful engagement with movement across diverse contexts. Such a perspective may help explain why PL showed a closer association with WB than PA in the present structural model.

These findings highlight the importance of considering both behavioral and psychosocial dimensions when examining the relationship between movement and WB in university populations.

### 4.1 Practical implications

The present findings have several practical implications for health promotion and physical education in higher education settings. First, approaches aimed at fostering PL may represent a promising strategy for supporting both active lifestyles and psychological WB among university students (38, 39). In practice, universities may consider embedding PL-oriented content into credit-bearing physical education courses, particularly courses designed to enhance confidence, self-reflection, and meaningful engagement in movement rather than focusing exclusively on performance outcomes (38, 39).

Second, the findings suggest the value of developing campus wellness programs that promote not only participation in PA but also students' perceived competence, autonomy, and understanding of movement. For example, university could offer non-competitive movement opportunities such as recreational walking programs, beginner-friendly activity classes, or informal group-based exercise sessions that reduce performance pressure and increase accessibility for students with diverse movement backgrounds (38, 39).

Third, reflective movement education may be especially useful in university settings. Programs that encourage students to recognize the personal meaning, enjoyment, and health value of movement may help strengthen the psychological dimensions of PL that are more closely associated with WB. Such approaches may be incorporated into general education courses, co-curricular health promotion initiatives, or student support services that aim to promote holistic development (38).

Finally, universities may benefit from creating campus environments that facilitate accessible and flexible opportunities for PA throughout the academic day. Examples include walking-friendly campus design, open recreational spaces, and low-barrier opportunities for movement participation. These strategies may help position PL as part of a broader student development agenda that integrates physical, psychological, and education WB (38, 39).

### 4.2 Limitations and future directions

Several limitations of this study should be acknowledged. First, the cross-sectional design precludes causal inference regarding the relationships among PL, PA, and WB. Although causal relationships cannot be established, the SEM framework allows for the examination of theoretically grounded associations among the constructs. Longitudinal and intervention-based studies are needed to clarify temporal ordering and potential reciprocal relationships among these variables (7, 40).

Second, the study relied on self-report measures, which may be subject to recall bias or social desirability bias. This issue may be particularly relevant for PL and WB, which involve self-perception and subjective evaluations. Future studies may benefit from incorporating objective PA measures and mixed-method approaches to capture both behavioral and experiential aspects of movement participation (27).

Third, the sample was drawn from a single university in South Korea, which limits generalizability of the findings. Multi-institutional and cross-cultural studies are needed to examine whether the observed relationships are consistent across different educational and sociocultural contexts. Future research should test model invariance across demographic and behavioral subgroups.

Finally, given the non-significant PA-WB pathway observed in the present study, future research should explore potential mediators and moderators that may influence the relationship between PA and WB. Factors such as motivation quality, perceived competence, environmental constraints, and social context may help explain the conditions under which PA is associated with psychological WB (21, 22).

## 5 Conclusion

The present study found that PL was positively associated with both PA and WB among South Korean university students, and that PL showed a significant direct association with WB after accounting for PA in the structural model. These findings suggest that PL may function not only as a correlate of active behavior but also as a psychosocial resource that supports WB during emerging adulthood (7, 18). Although the structural relationships among PL, PA, and WB require further clarification through longitudinal research, the present findings indicate that PL may represent a promising target for student health promotion in higher education settings.

## Data Availability

The raw data supporting the conclusions of this article will be made available by the authors, without undue reservation.
